# Budget impact analysis of breast cancer medications: a systematic review

**DOI:** 10.1186/s40545-022-00493-1

**Published:** 2022-12-29

**Authors:** Ghader Mohammadnezhad, Melika Sattarpour, Najmeh Moradi

**Affiliations:** 1 School of Pharmacy, Shahid Beheshti University of Medical Sciences, Tehran, Iran; 2 Health Management and Economics Research Center, Health Management Research Institute, Iran University of Medical Sciences, Tehran, Iran

**Keywords:** Budget impact analyses, Anti-cancer medication, Breast cancer, Pharmacoeconomics, Budget holders

## Abstract

**Background:**

Breast cancer (BC) is the most common cancer globally among women, with 2,261,419 new cases in 2020; systemic treatment may be neo-adjuvant, adjuvant, or both. BC subtype guides the standard systemic therapy administered, which consists of endocrine therapy for all HR + tumors, trastuzumab-based HER2-directed antibody therapy plus chemotherapy for all HER2 + tumors (with endocrine therapy given in addition, if concurrent HR positivity), and chemotherapy alone for the triple-negative subtype. This study aimed to identify, evaluate, and systematically review all budget impact analyses (BIAs) of BC medications worldwide.

**Methods:**

PubMed, Scopus, and Web of Science Core Collection databases were thoroughly searched up to 26th March 2022 to identify original published studies which evaluate BIA of BC medications. ISPOR Task Force guidelines were used to assess the quality of included studies. This study was conducted and reported following the Preferred Reporting Items for Systematic Reviews and Meta-Analyses (PRISMA) guidelines.

**Results:**

In total, 17 BIAs were included in the study. About half of the studies were conducted in Europe. The results of the BIAs showed that most of the included BIAs are conducted from the payer’s perspective; they have different methodological frameworks for recommended chemotherapy, targeted therapy, and immunotherapy agents to treat BC. For the same medications, the results of budgetary effects are not consistent in diverse countries. Nine out of the 17 studies were focused on trastuzumab, in which the biosimilar form reduced costs, but the brand form increased costs, especially in a 52-week treatment period.

**Conclusion:**

Researchers should conduct the budget impact analysis of high-value medications such as anti-tumor drugs more objectively, and the accuracy of parameters needs to be more strictly guaranteed. Furthermore, it is worthy of declaring that the budgetary impact of the same drug is not always consistent over time, so the researchers should measure access to medication in the long run.

## Background

Breast cancer (BC) is the most incident cancer globally among women, with 2,261,419 new cases in 2020; its incidence and prevalence worldwide are increasing, and it is the fifth leading cause of death due to cancer in women [1, 2]. Unlike women, breast cancer in men has been less evaluated and studied. According to the American Cancer Society data, in 2020, 2620 new BC cases in men were identified in the United States [3–5]. Although the number of new cases has increased in recent decades, the rate of metastatic cases and deaths from BC has decreased with increasing knowledge of screening, early diagnosis, monitoring, and discovery of new drugs, especially in developed countries [6–9].

Generally, screening of high-risk individuals and targeted BC treatment is performed by receptors on the surface of the breast neoplasms. Targeting the estrogen and progesterone receptors and the human epidermal growth factor receptor-2 (HER2) are widely used to prevent and treat BC [10, 11]. For non-metastatic BCs, the main treatment goals are to eradicate the tumor from the breast and lymph nodes in the area and prevent metastatic occurrence [12, 13]. Systemic treatment may be pre-surgical (neo-adjuvant), post-surgical (adjuvant), or both. BC subtype guides the standard systemic therapy administered, which consists of endocrine therapy for all HR + tumors (with some patients requiring chemotherapy as well), trastuzumab-based HER2-directed antibody therapy plus chemotherapy for all HER2 + tumors (with endocrine therapy given in addition, if concurrent HR positivity), and chemotherapy alone for the triple-negative subtype. For metastatic BC, therapeutic aims are increasing life years and relieving symptoms. The same basic categories of systemic treatment are used in metastatic BC as the neo-adjuvant/adjuvant approaches. The treatment process in these patients is long term and complicated, which imposes high costs on the healthcare systems [14–17].

Budget impact analysis (BIA) estimates the economic consequences of adopting a new intervention in a health system. Significantly, such analyses predict how the change will affect the combination of drugs and other treatments used to trace health costs in those conditions [18–20]. Unlike cost-effectiveness analysis (CEA), which measures the value of new interventions in financial elements/additional units of health benefits, the BIA aims to assess the affordability of health interventions is a concern for health policy-makers. Therefore, the BIA should complement the CEA and, as a parallel task, decide on the best way to allocate budgets appropriately [21–23].

This study is the first systematic review aimed to evaluate and review all BIAs of BC medications in recent 20 years.

## Materials and methods

### Study overview

Following Preferred Reporting Items for Systematic Reviews and Meta-Analyses (PRISMA), in this study, we systematically reviewed the budget impact of medications recommended in different types and stages of BC worldwide.

### Search strategy

Based on the published criteria for BIA studies, a systematic search query was made to identify related articles in PubMed, Scopus, and Web of Science databases. The original papers, published up to 26th March 2022, evaluated the budget impact as the primary or secondary outcome and were identified and saved in the reference manager. There was no time or language limit to include articles in the study. The following query was used: [(“budget impact*” OR “budgetary*” OR “budget impact analysis” OR “budget impact study” OR “financial impact” OR “economic impact”) AND (“breast cancer*” OR “breast neoplasm” OR “breast malignancy” OR “breast carcinoma”)].

### Eligibility

The inclusion criteria were: 1. budget impact articles, 2. articles that analyzed cost-effectiveness along with budget impact, 3. studies that looked at BIA of BC drugs. Records evaluating cancer diagnostic and screening methods evaluated budget impact for non-BC medications, economic evaluations lacking BIA, and all non-economic articles were excluded. Comments, letters to the editor, and conference abstracts were also excluded.

### Article selection

First, duplicate records were identified and deleted. After ensuring that any record was unique, titles and abstracts were screened concerning the subject of the current review. The screened studies were analyzed in terms of full-text eligibility and entered into the study. The whole process was rechecked by another researcher and finally documented in a PRISMA flow chart.

### Data extraction

According to the ISPOR Task Force guidelines, a characteristics table was developed to mention the main items of the studies, including the first author’s name, population size, population features, clinical data, publication year, intervention(s), comparator(s), budget holder’s perspective, time horizon, discounting, market share per year, cost calculations, sensitivity analysis. Then, the findings of all included articles on different aspects of BIA studies, such as treatment strategy, included cost, market share, and budget impact value, were extracted, summarized, classified, and interpreted.

### Quality assessment

The compliance and quality level of the included studies to the ISPOR Task Force guidelines was assessed and rechecked by another author [24, 25]. This task force developed expert consensus guidance on international good practice standards in health economic research and the use of this research in decision-making by healthcare providers. Nine items are evaluated according to this guideline, including study, target population, time horizon, hypothetical scenario, comparator, framework description, data collection, validation, and sensitivity analysis.

Studies that clearly stated at least seven items were recognized as quality BIA studies.

## Results

### Characteristics of included studies

**Fig. 1:**
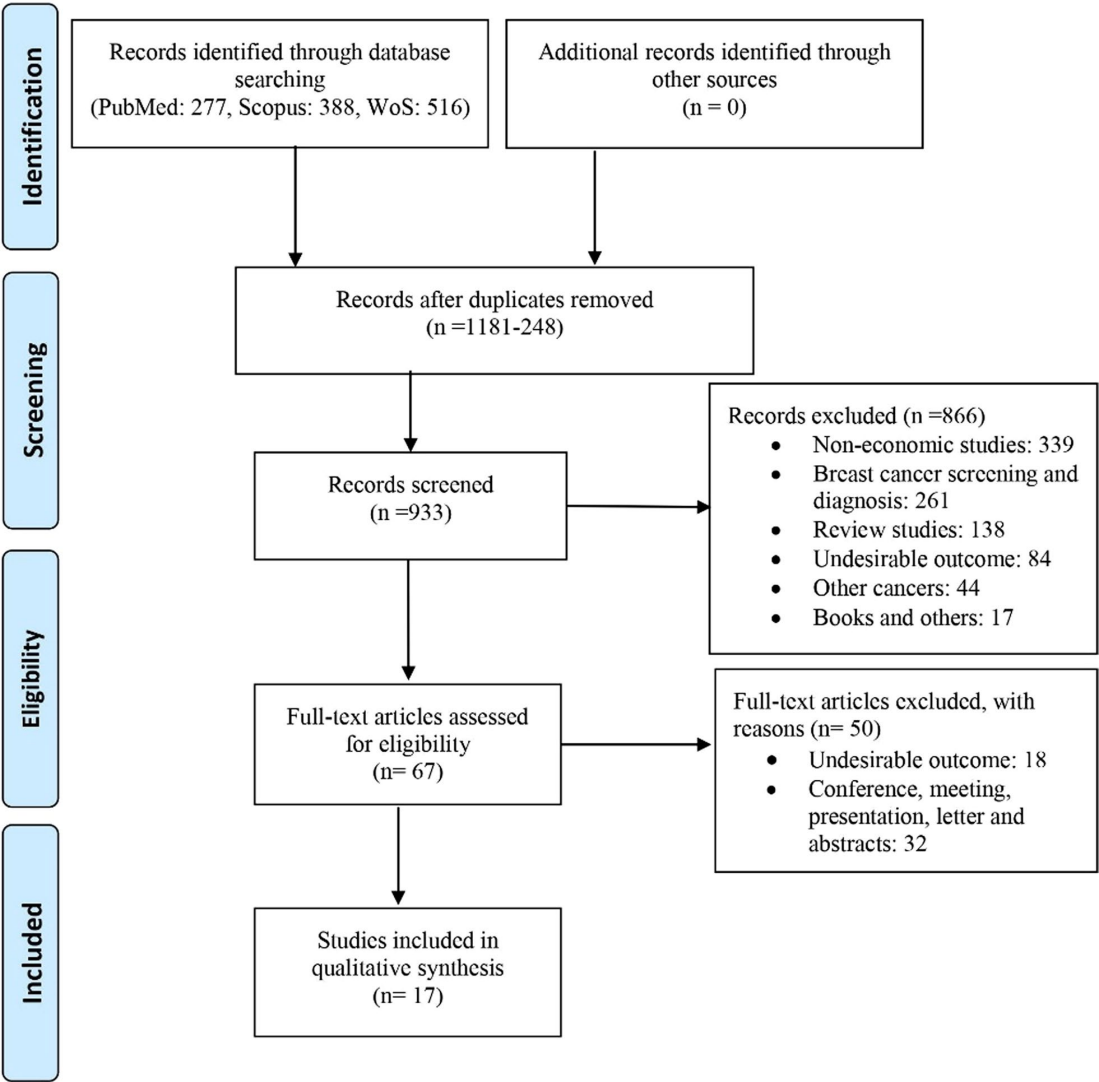
PRISMA flow diagram

The search records in the three databases were 1181, which remained at 933 after the duplicates were removed. Eight hundred sixty-six records were excluded following the systematic review’s aim, and the remaining 67 were screened for full-text access. Of these 67 records, 50 were removed for reasons, and 17 BIAs (15 in English and 2 in Russian) were systematically reviewed. The search process and exclusion details are demonstrated in Fig. 1.

Table 1 shows the general characteristics of the BIAs included in the study. Among them, about half (*n* = 8) of the studies were conducted in Europe [26–33], three in the United States [34–36], three in Asia [37–39], two in Russia [40, 41], and one in Australia [42]. BIAs were performed in the period 2004–2021. Twelve studies explicitly mentioned their funding source; two mentioned they did not have any funding source [26, 33], and three did not disclose this part [32, 40, 42]. All but one study [39] cited the study perspective, two of which used the social perspective study [27, 38] and more studies from the payer or public health care perspective. All studies, except for one case based on real-world data [27], were based on modeling. Nine studies only evaluated the budget impact of anti-BC interventions, eight model-based studies evaluated the cost-effectiveness of the drug and two Russian studies in the form of cost-minimization [40, 41].

**Table 1:** Study characteristics

Study	Year	Country	Funding	Perspective	Study type	Study base	Intervention	Comparator	Population	Population size
Ivanov et al. [40]	2021	Russia	N.R	Federal Compulsory Health Insurance Fund	CMA + BIA	Model	PO vinorelbine	Ixabepilone	Metastatic BC	1000 hypothetical patients
Elsamany et al. [37]	2020	Saudi Arabia	Roche Products Saudi Arabia	Governmental health sector	BIA only	Model	SC trastuzumab	IV trastuzumab	HER 2 + BC	394 newly diagnosed cases/ year
Pouwels et al. [27]	2020	Netherlands	Netherlands Organization for Health Research and Development, Eisai, Novartis BV, Roche, Pfizer and Eli Lilly	Dutch societal	CEA + BIA	Real-world	Eribulin	Non-eribulin chemotherapy	The Southeast Netherlands advanced BC registered patients	–
Genuino et al. [38]	2019	Philippines	Mahidol University and the International Decision Support Initiative (iDSI)	Healthcare system and societal	CUA + BIA	Model	Adjuvant trastuzumab	Standard chemotherapy alone	HER 2 + early-stage BC	64,046
										Prevalent
										Cases in 5 years, 21,057
										New cases (23.17% HER 2 + , 80% early stage)
Avxentyev et al. [41]	2019	Russia	Pfizer	Russian healthcare system	CMA + BIA	Model	CDK 4/6 inhibitor + letrozole	Another CDK 4/6 inhibitor + letrozole	HR + HER 2 -advanced BC	8098–8221 newly diagnosed patients/ year
Lee et al. [28]	2019	28 European Countries	Chung-Ang University research	Payer	BIA only	Model	Biosimilar Trastuzumab (CT-P6)	Originator trastuzumab	HER 2 + BC	Early BC: 79,723 (2018) Metastatic BC: 14,844 (2018)
Mistry et al. [34]	2019	USA	Novartis	US payer	BIA only	Model	Ribociclib + letrozole	Letrozole alone, palbociclib + letrozole, fulvestrant + anastrozole, fulvestrant alone, exemestane, tamoxifen, anastrozole alone, palbociclib + Fulvestrant, Fulvestrant + letrozole, and eribulin	Post-menopausal women with HR + /HER2 -advanced /metastatic BC	263 cases in a cohort of 1 million members at first year and increase in subsequent years
Clarke et al. [33]	2017	UK	No fund	English NHS payer	CEA + BIA	Model	Different durations of adjuvant	–	Early BC	–
							trastuzumab			
Cesarec et al. [26]	2017	Croatia	No fund	Public healthcare	BIA only	Model	Biosimilar Trastuzumab	–	BC	479 cases in 1 year
Lewis et al. [39]	2015	Kazakhstan	Novartis Pharma AG	N.R	BIA only	Model	Everolimus + letrozole/anastrozole	Letrozole/anastrozole,	Post-menopausal HR + HER2 -advanced BC	776 prevalent, and 133, 145, 157, and 170 newly diagnosed in 2015–2018
								Chemotherapy,		
								Tamoxifen		
Benjamin et al. [30]	2013	France	GSK	French National Health Insurance	BIA only	Model	Trastuzumab-based therapy	Lapatinib + capecitabine	HER2-positive metastatic BC	4182 cases (73% trastuzumab-based therapy, 27% lapatinib + capecitabine)
Xie et al. [43]	2013	US	Novartis	US payer	BIA only	Model	Exemestane + everolimus	Exemestane Fulvestrant Tamoxifen	HER2-positive advanced BC	72 cases / 1 million receiving their first treatment after letrozole or anastrozole failure, and 159 cases / 1 million receiving a second treatment after failing letrozole or anastrozole and the first treatment
Purmonen et al. [31]	2010	Finland	Finnish Office for Health Technology Assessment (Finohta)	Single hospital district	BIA only	Model	Trastuzumab	–	Early BC	86.6/100,000 in 2006
Ho et al. [36]	2009	US	Bristol-Myers Squibb Company (BMS)	Payer	BIA only	Model	Ixabepilone	Various anti-cancer medications	Treatment-resistant metastatic BC	570 cases/1 million
Neyt et al. [32]	2008	Belgium	N.R	Payer	CEA + BIA	Model	Trastuzumab	ST	Early BC	–
Millar et al. [42]	2007	Australia	N.R	Australian health system	CEA + BIA	Model	Trastuzumab	ST	Early BC	–
Marchetti et al. [29]	2004	Italy	AstraZeneca SpA	Italian National Health Service	CUA + BIA	Model	Third-Generation Aromatase	Tamoxifen, megestrol, paclitaxel, docetaxel, vinorelbine	Advanced BC	–
							Inhibitors			

Nine studies dealt with the administration methods or course of treatment or the branded drug or biosimilar trastuzumab [26, 28, 30–33, 37, 38, 42], and of the other eight studies, two studies dealt with the BIA of everolimus [35, 39] and two with cyclin-dependent kinase 4/6 inhibitors (CDK4/6i) in combination with letrozole [34, 41]. Only one study did not identify the type of BC and its stage [26]. While 14 articles mentioned the BC stage (nine cases of metastatic BC or advanced BC and five cases of early stage BC) and ten articles mentioned that their target population is patients with HER-2 positive BC [26, 28, 30–33, 37, 38, 42, 43], and three articles mentioned that their target population is HER-2 negative [34, 39, 41]. In general, the eligible population has been mainly due to the indication of the under-research drug or the coverage of the study perspective. Eleven articles mentioned the target population and how to calculate it, and six articles did not explicitly mention it or use hypothetical populations [27, 29, 32, 33, 40, 42]. Thirteen articles stated that none of the article's authors had a conflict of interest, and four did not say it clearly [29, 31, 32, 42].

### Methodology of included studies

Table 2 summarizes the methodology of BIA studies. Six out of 17 studies included the budget impact model [26, 28, 30, 35–37], five Markov studies [29, 33, 38, 41, 42], two cohort-based studies [34, 39], and other studies based on health-state modeling [27], health economic model (HEM) [32], and spread-sheet [31], and one study did not state it clearly [40]. All studies expressed their time horizon between 1 and 5 years, and four studies used lifetime [32, 33, 38, 42]. Only in model five studies the discount rate was expressed and used (3–4%) [27, 29, 30, 33, 42], and 12 studies did not express it or use it in BIA.

**Table 2:** Methodology and BIA results

Study	Model structure	Time horizon	Discounted rate (per annum)	Treatment strategy	Included costs	Market share	Sensitivity analysis	Incremental budget impact value	Result
[40]	N.R	1 year	No	PO vinorelbine vs. ixabepilone	Drug cost	N.R	One-way	18,355,044 rubles for the budget fund	The use of oral vinorelbine provides financial savings for a medical organization
[37]	BIM	3 years	No	SC vs. IV trastuzumab	Drug cost, preparation and administration, indirect cost of patient productivity loss	1. Gradual Replacement: 25, 50, 75%	One-way	Total incremental costs/ 3 years: -34,527,346 SAR (USD19,181,858) in scenario 1,—69,054,692 SAR (USD36,363,717) in scenario 2	SC trastuzumab was budget saving
						2. Total Replacement: 100%			
[27]	Partitioned survival model	5 years	4%	Eribulin vs. non eribulin chemotherapy	Drug chemotherapy cost, weekly monitoring, radiotherapy, hospitalization, surgery	N.R	One-way and probabilistic	Annual budget impact was M€1.9 and the annual risk of reimbursing eribulin was M€2.7	N.R
[38]	Markov	Lifetime	3.50%	Adjuvant trastuzumab vs. ST	DMC of adjuvant trastuzumab, Drugs and CT, administration, cardiac function assessment, hospital admission, echocardiography, drugs, cardiac monitoring	N.R	One-way and two-way	1st year: 13,909 MPHP, other fiscal four years: 2946, 2509, 2110, 1910 MPHP, respectively	That markedly transcends the usual annual budget for the procurement of all BC drugs covered under the DOH BCMAP
[41]	Markov	5 years	No	Palbociclib + letrozole vs. ribociclib + letrozole	Medications, adverse event treatments	N.R	One-way	9 087 million RUB or 22.5%, saving	Using palbociclib is the cost-saving for post-menopausal women with locally advanced or metastatic HR-positive HER2-negative BC, compared to ribociclib
[28]	BIM	5 years	No	Biosimilar trastuzumab vs. originator	Drug price	25% annual market share over the 5 years	One-way	1. 20% in 1st year, switching growth of 5, 10, and 15% saved the total budget of M€1134, 1507, and 1881, respectively	3% budget saving
								2. 30% in 1st year, switching growth of 5, 10, 15% saved the budget of M€1514, 1887, 2261, respectively	
								3. 40% in 1^st^ year, switching growth of 5 and 10% saved M€1894 and 2267, respectively	
[34]	Cohort-based BIM	3 years	No	Ribociclib + letrozole vs. other scenarios mentioned in Table 1	Wholesale acquisition cost, administration, diarrhea, fatigue, infection, nausea. febrile neutropenia, pulmonary embolism, vomiting, anemia	3.3%, 11.9%, 19.3% for years 1 through 3	One-way	$3.01 M over three years	Cost-saving first-line treatment option
[33]	Markov	Lifetime	3.50%	9 weeks vs. 52 weeks trastuzumab therapy	Drug, monitoring, recurrence and death	N.R	One-way and Bayesian probabilistic	£132 million reduction in costs	Adjuvant trastuzumab was cost-saving in England 2014
[26]	BIM	1 year	No	Biosimilar scenario 1, based on all approvals in	Drug acquisition,	N.R	One-way	1. 15% drug price discount: scenario 1: €294,940	Significant drug cost savings
				2015, and biosimilar scenario 2, based on approvals after				scenario 2: €260,436	
				15-Feb				2. 25%: scenario 1: €491,958 scenario 2: €434,453	
								3. 35%: scenario 1: €688,977	
								scenario 2: €608,470	
[39]	Cumulative cohort model	5 years	No	Everolimus + letrozole/anastrozole vs. letrozole/anastrozole, chemotherapy, tamoxifen	Hormone therapy, radiotherapy, adverse events, outpatient and inpatient services, lab test, examination, imaging, additional therapy	1% in the first year, 2% in the second year, and 3% in the third, fourth, and fifth	One-way	Incremental impact of introducing everolimus in 5 years: 201,359,752 tenge	2.69% increase in total cost, which was modest
[30]	BIM	3 years	No	TBT vs. lapatinib + capecitabine	Physician consultation, medical examination, transportation, administration, drug cost	Increases linearly over the time horizon	One-way and exploratory	€129,519,134 in 2012, €123,254,493 in 2013, €116,790,315 in 2014	The potential savings for Health Insurance with the use of oral drug due to the reduction of outpatient hospitalizations
[43]	BIM	1 year	No	Exemestane + everolimus vs. Exemestane,	Wholesale drug cost, Co-payment per fill, dispensing fee per fill	10%	One-way and two-way	Incremental budget saving: $522,336	A modest net increase in total budget
				Fulvestrant, tamoxifen,					
[31]	Spreadsheet model	4 years	No	Adjuvant trastuzumab vs. ST	Drug cost	N.R	One-way and probabilistic	€ 1.30	The trastuzumab acquisition costs were partially offset by the reduction in costs associated with the treatment of cancer recurrence and metastatic disease
								Million	
[36]	BIM	3 years	No	1. Ixabepilone monotherapy in ATC-p 2. Ixabepilone + capecitabine in AT-p patients	Drug cost	1. ATC-p: 8.39, 8.64, 8,61% for years 1 Through 3 2. AT-p: 2.09, 2.03, 2.00% for years 1 through 3	One-way and multivariate	1. ATC-p:	A relatively minimal incremental budget impact 9-week therapy was more cost-saving
								$41,428,	
								$47,236,	
								$47,695 for 3 consecutive years	
								2. AT-p: $23,103, $22,287, $21,943 for 3 consecutive years	
[32]	HEM	Lifetime	No	Adjuvant trastuzumab for 9 and 52 weeks vs. one-year non-trastuzumab therapy	Drug cost, MBC treatment cost, heart failure, administration, local recurrence, follow-up	N.R	One-way and multi-way probabilistic	5.17 M€ for 597 patients in 9 weeks trastuzumab therapy, 19.96 M€ for 491 patients in 52 weeks trastuzumab therapy	
[42]	Markov	Lifetime	3%	Adjuvant trastuzumab vs. ST	Metastatic disease, local recurrence, heart failure, treatment of diseases other than BC,	N.R	One-way and multiple univariate	The additional net cost was $A7.3 million in each 1000-patient-cohort	N.R
[29]	Markov	3 years	3%	Anastrozole and letrozole vs. tamoxifen	Hormone therapy, radiotherapy, chemotherapy, antianemics, antiemetics, pain medications, doctor consultations, hospitalization, homecare assistance,	N.R	One-way	€15 million	12% budget increase

In nine studies, trastuzumab was studied as one of the budget holder arms of the BIA. In these studies, the use or non-use [30–32, 38, 42], originator form or biosimilar [26, 28, 37], and 9-week or 52-week period of trastuzumab-based therapy were investigated [32, 33]. Two studies examined the budget impact of everolimus in HR + patients [35, 39], and two studies examined the inclusion of cyclin-dependent kinase (CDK) 4/6 inhibitor drugs in the drug list of the countries [34, 41]. Other studies evaluated the budget impact of vinorelbine [40], ixabepilone [36], and third-generation aromatase inhibitors [29].

The assumed costs in all studies included at least the drug cost. Some studies have suggested other costs for treatment with new drugs. Six studies included the cost of side effects and their management, including heart failure with trastuzumab, in the cost input [29, 32, 34, 38, 39, 41, 42]. In addition to drug costs and side effects, studies included the cost of prescribing, preparation, dispensing, transport, and productivity loss. Contrary to the cost input, which was clearly stated in all studies, the market share trend was mentioned in only seven cases. This trend was expressed in different scenarios, such as the immediate replacement of the old drug with the new drug, the linear replacement trend, the increasing annual trend of new drug consumption until complete replacement, and the increasing trend to capture a part of the old intervention market.

Of all the BIA studies included in the systematic review, only two studies did not express the final result of adding the drug to the breast cancer patients’ medication regimen [27, 42], but in other studies, the final result was explicitly reported in the form of incremental budget impact value and total annual cost.

### Quality assessment

Table 3 summarizes the ISPOR task force guidelines of the included BIAs [24]. The results of the quality assessment of studies according to this guideline showed that all articles entered in the systematic review are of high quality and have the necessary items for a BIA. All studies reported the time horizon, BIA framework, data collection, and sensitivity analyses. One study did not declare the perspective [39], six studies did not declare the target population estimation [27, 29, 32, 33, 40, 42], two studies did not declare their hypothetical scenario [29, 38], three did not mention the comparator [26, 31, 33], and eight studies did not validate their model and results [26, 31, 32, 34, 36, 39–41].

**Table 3:** Quality assessment results

Study	Perspective	Target population estimate	Time horizon	Hypothetical scenario	Comparator	Framework description	Data collection	Validation	Sensitivity analysis	Total (%)
Ivanov et al. [40]	✓		✓	✓	✓	✓	✓		✓	77.8
Elsamany et al. [37]	✓	✓	✓	✓	✓	✓	✓	✓	✓	100
Pouwels et al. [27]	✓		✓	✓	✓	✓	✓	✓	✓	88.9
Genuino et al. [38]	✓	✓	✓		✓	✓	✓	✓	✓	88.9
Avxentyev et al. [41]	✓	✓	✓	✓	✓	✓	✓		✓	88.9
Lee et al. [28]	✓	✓	✓	✓	✓	✓	✓	✓	✓	100
Mistry et al. [34]	✓	✓	✓	✓	✓	✓	✓		✓	88.9
Clarke et al. [33]	✓		✓	✓		✓	✓	✓	✓	77.8
Cesarec et al. [26]	✓	✓	✓	✓		✓	✓		✓	77.8
Lewis et al. [39]		✓	✓	✓	✓	✓	✓		✓	77.8
Benjamin et al. [30]	✓	✓	✓	✓	✓	✓	✓	✓	✓	100
Xie et al. [43]	✓	✓	✓	✓	✓	✓	✓	✓	✓	100
Purmonen et al. [31]	✓	✓	✓	✓		✓	✓		✓	77.8
Ho et al. [36]	✓	✓	✓	✓	✓	✓	✓		✓	88.9
Neyt et al. [32]	✓		✓	✓	✓	✓	✓		✓	77.8
Millar et al. [42]	✓		✓	✓	✓	✓	✓	✓	✓	88.9
Marchetti et al. [29]	✓		✓		✓	✓	✓	✓	✓	77.8
Total (%)	94.1	64.7	100	88.2	82.4	100	100	52.9	100	86.9

## Discussion

In this study, we systematically reviewed all BIAs of anti-breast cancer drugs in the world for the first time. These studies were primarily performed in developed countries for effective and expensive breast cancer drugs such as trastuzumab. We tried to conclude the impact of breast cancer drugs on the health system budget. One of the most important points we found in this systematic review was that despite valid global and national guidelines, there is a great deal of heterogeneity in BIAs in model design, results reporting and results in validation. Many countries, including France, Ireland, India, and Canada, have national guidelines for reporting BIA study results [44–48]. However, BIA studies are not yet developed in a uniform and specialized form. In the quality assessment studies included in the systematic review by ISPOR task force guideline, we found that the central part that researchers have neglected is validation [24], which has not been reported in half of the studies. However, the reporting quality of different parts of a BIA was more promising in this study compared to previous systematic reviews [49, 50]. One of the problems of BIAs is that due to the cooperation and support of pharmaceutical companies in these studies and cost-effectiveness studies, the results of the studies may be subject to biases to the satisfaction of pharmaceutical companies. Researchers may also be reluctant to publish unfavorable findings for themselves and pharmaceutical companies. Therefore, it is recommended that the causes of bias and non-reporting of findings be listed, minimized, or eliminated in the BIA.

Another thing to keep in mind is that BIAs targeted for the inclusion of drugs on the drug list of countries or BIAs reported with cost-effectiveness analyses may be more inclined to present the new drug as a cost-saving strategy.

Another critical issue is the time horizon of BIA studies. It should also be noted that increasing or decreasing costs in the short term should not be the basis for the entry and exit of drugs from the drug list. A long-term budget impact must be made to judge reducing or increasing costs properly. For example, new medications with sound effects and high prices usually increase the cost of treatment. While in the long term, these drugs can reduce treatment costs. Through the long-term budget time horizon studies, the increase or decrease in budget trend could be seen to predict whether funding was affordable or not. To make better use of BIA studies, the focus should be on expensive cancer drugs that impose a more significant economic burden on the health system and subtypes of breast cancer that have a higher incidence rate in different communities.

This study has several strengths, including coverage of non-English languages (avoidance of tower of babel bias) [51], no time limit, and comprehensive coverage of data reported in BIA studies. At the same time, the present study has its limitations. Some scientific databases, such as EMBASE, were not available for use in the search. The results of various studies were not comparable due to differences in the currency used and the time difference.

## Conclusion

In conclusion, the results of the BIAs showed that most of the included BIAs are conducted from the payer’s perspective; they have different methodological frameworks for recommended chemotherapy, targeted therapy, and immunotherapy agents to treat BC. For the same medications, the results of budgetary effects are not consistent in a diverse country. Researchers should conduct the budget impact analysis of high-value medications such as anti-tumor drugs more objectively, and the accuracy of parameters needs to be more strictly guaranteed. The high-quality BIAs should be based on real-world data to provide reliable results for policy-makers. Furthermore, it is worthy of declaring that the budgetary impact of the same drug is not always consistent over time, so the researchers should measure access to medication in the long run.

## Data Availability

Not applicable.

## Abbreviations

BC: Breast cancer
BIA: Budget impact analysis
HER2: Human epidermal growth factor receptor-2
CEA: Cost-effectiveness analysis
HR: Hormone receptor
PRISMA: Preferred Reporting Items for Systematic Reviews and Meta-Analyses
HEM: Health economic model
CDK4/6i: Cyclin-dependent kinase 4/6 inhibitors
